# Comparison of Creatinine and Cystatin C to Estimate Renal Function in Geriatric and Frail Patients

**DOI:** 10.3390/life12060846

**Published:** 2022-06-07

**Authors:** Erik Dahlén, Linda Björkhem-Bergman

**Affiliations:** 1 Jakobsberg Geriatric Clinic, Jakobsberg’s Hospital, Järfälla, 177 31 Stockholm, Sweden; 2 Division of Clinical Geriatrics, Department of Neurobiology, Care Sciences and Society, Karolinska Institutet, Blickagången 16, Neo Floor 7, Huddinge, 141 83 Stockholm, Sweden; linda.bjorkhem-bergman@ki.se

**Keywords:** creatinine, cystatin C, frail elderly, geriatrics, glomerular filtration rate, renal insufficiency

## Abstract

The aim of this study was to compare estimated glomerular filtration rate (eGFR) with creatinine (eGFR_crea_) and cystatin C (eGFR_cys_) in geriatric and frail patients. A retrospective, cross-sectional study was performed at a geriatric clinic in Stockholm (*n* = 95). The revised Lund–Malmö equation was used to calculate eGFR_crea_ and the Caucasian-Asian-Pediatric-Adult (CAPA) equation was used for eGFR_cys_. The absolute mean percentage difference between eGFR_crea_ and eGFR_cys_ was used as a surrogate measure for accuracy in eGFR. Other outcome measures were consistency expressed in Lin’s concordance correlation coefficient and the proportion of consistent staging of renal failure. Subgroup analyses were performed with regard to frailty (according to Clinical Frailty Scale) and age. eGFR_cys_ estimated lower GFR than eGFR_crea_ across the entire study population as well as in all subgroups (*p* < 0.05). Difference between the estimates increased with increasing frailty (*r*^2^ = 0.15, *p* < 0.01), but was not significantly affected by age (*r*^2^ = 0.004, *p* = 0.55). In conclusion, eGFR_cys_ was significantly lower compared to eGFR_crea_ in geriatric and frail patients. Moreover, frailty had greater impact than age on the accuracy of eGFR. However, this study cannot determine if any of the estimates are preferable over the other in this patient group.

## 1. Introduction

Knowledge of patients’ renal function is of paramount importance in patient safety, especially for assessing renal ability to eliminate drugs. Kidney function is described by glomerular filtration rate (GFR), i.e., the volume of fluid filtered out of plasma through glomeruli per minute. Normal GFR is 100–130 mL/min [1]. From the age of 40–50 years, there is a gradual decline in renal function as part of normal ageing [1]. By the age of 80, GFR is expected to have decreased by 50% [2]. Frail elderly people are a vulnerable group due to polypharmacy and are at increased risk of adverse drug reactions [3]. In Sweden, approximately 10% of emergency admissions of elderly people are due to adverse drug reactions, 60% of which are possibly avoidable [4]. Frailty is a consequence of biological ageing, but not all older people are frail [5].

GFR can be measured (mGFR) by administering an *exogenous* marker intravenously, e.g., iohexol, and calculating the elimination rate of the substance by a follow-up urine or blood sample. However, this is time consuming and resource intensive and is only used when a precise estimation of renal function is necessary, e.g., before chemotherapy or kidney donation [1]. In clinical practice, *endogenous* markers are measured instead, using mathematical equations to give an estimated GFR (eGFR) without the need to measure elimination rate. eGFR can be calculated in absolute numbers or relative to a standardized body surface area of 1.73 m^2^. Relative eGFR is used for assessing degree of renal impairment (Table 1) and absolute eGFR is used for dosage of drugs [1].

Creatinine is the most common endogenous marker used to calculate eGFR (eGFR_crea_). It is a break-down product from muscle tissue, and plasma levels are influenced by muscle mass, meat intake but also dehydration [1]. Low muscle mass, sarcopenia, is common in the elderly [7], but is to a higher extent associated with frailty [8,9]. *Sarcopenic obesity* is not uncommon in this patient group [10]. In these circumstances, BMI becomes a blunt measure of muscle mass.

Cystatin C is an alternative endogenous protein used to estimate renal function (eGFR_cys_). It is a protease inhibitor produced by all nucleated cells and is not affected by muscle mass [1,2], but may be affected by other factors including hypo- and hyperthyroidism (falsely decreased and falsely elevated, respectively) [1,2] and high-dose steroid therapy (falsely elevated) [1,11]. In Sweden, Cystatin C is approximately seven times more expensive to analyze compared to creatinine.

The best estimate of GFR is, however, obtained by calculating the mean of eGFR_crea_ and eGFR_cys_ (eGFR_crea+cys_), alternatively from composite equations using both markers [1,12,13,14,15,16,17,18,19].

In 2012, the Swedish Council on Health Technology Assessment (SBU) published an extensive systematic review on methods to estimate and measure renal function (1). They concluded that creatinine and cystatin C equations are equivalent in younger patients, but evidence was lacking in the elderly population. Since then, several studies on different equations have shown that eGFR_crea_ and eGFR_cys_ are equivalent also in the elderly [12,14,15,16,18,20,21,22,23,24]. However, a majority of the studies have been conducted on patients referred for GFR measurement, patients connected to nephrology clinics or on large study cohorts in an outpatient setting. Frail elderly people represent the majority of patients in geriatric wards [25,26,27]. Increasing frailty predisposes risks for inpatient care [26,28]. GFR measurement is rarely indicated in these patients. This might explain why the geriatric context is sparsely represented in the literature. After SBU’s extensive report, two studies have been conducted in geriatric clinics globally, which compare eGFR_crea_ and eGFR_cys_ with mGFR [20,29]. Another study has been conducted in a nursing home but did only relate eGFR_crea_ with eGFR_cys_ without having mGFR as reference [30]. No previous study has compared eGFR_crea_ with eGFR_cys_ in frail patients in a geriatric inpatient clinic.

The aim of the present study was to compare eGFR_crea_ and eGFR_cys_ in frail patients in a geriatric inpatient clinic. The hypothesis was that eGFR_crea_ and eGFR_cys_ differ signifi-cantly from each other where Cystatin C estimates lower GFR compared to creatinine.

## 2. Materials and Methods

### 2.1. Study Design and Study Population

This is a retrospective, cross-sectional study at Jakobsberg Geriatric Clinic in Stockholm, Sweden. The clinic has a capacity of 90 beds and receives referrals for acute and tertiary geriatric care from community health centers and other hospitals in Stockholm County. During February and April 2021, all patients at a designated ward were screened with both eGFR_crea_ and eGFR_cys_ at admission as a part of a local quality improvement work. For this study, medical records were reviewed retrospectively in order to collect eGFR_crea_, eGFR_cys_ and descriptive data for each patient at admission during this period. The creatinine-based revised Lund–Malmö equation (LMR) [31] and the cystatin C-based Caucasian-Asian-Pediatric-Adult equation (CAPA) [32] are laboratory standards in the Stockholm County and were used for calculation of eGFR.

#### 2.1.1. Inclusion Criteria

The inclusion criteria were as follows:

- Both eGFR_crea_ and eGFR_cys_ available at admission.
- All diagnoses, sex and ages.

#### 2.1.2. Exclusion Criteria

The exclusion criteria were as follows:

- eGFR_cys_ > 90 mL/min. When eGFR_cys_ exceeded 90 mL/min, it was only reported as ‘>90 mL/min’ in the lab results. The statistical analysis would be skewed if these values were included.

No consideration was given to thyroid disease, high-dose steroid therapy or low weight in the development of LMR and CAPA. Therefore, these where *not* exclusion criteria in this study. Well-controlled hypo- or hyperthyroidism is unlikely to affect plasma levels of cystatin C [33]. Similar reasoning can be seen in other studies [12,18,34]. However, we controlled for these factors to detect any differences in the results.

Thyroid disease was defined as presence of thyroid treatment at admission (ATC code H03). High-dose steroids was defined as >0.170 mg/kg/day prednisolone equivalents at admission [11]. Low weight was defined as BMI < 20 kg/m^2^ (1), based on current height and weight at admission.

### 2.2. Data Acquisition

Descriptive data on age, sex, BMI, diagnosis (based on the 10th revision of The International Classification of Diagnosis and Related Health Problems by WHO, *ICD-10*), presence of thyroid disease or high-dose steroid therapy and stage of renal failure according to Kidney Disease Outcomes Quality Initiative (KDOQI) [6] were collected. However, no consideration was given to proteinuria and whether it was acute or chronic renal failure.

#### Laboratory Analyses

Blood samples of creatinine and cystatin C were collected at admission and were analyzed using Siemens ADVIA XPT. For creatinine, the enzymatic colorimetric method was used with Siemens ADVIA Chemistry Enzymatic Creatinine_2 reagent (traceable to the international reference material SRM967 from the National Institute for Standards and Technology). For cystatin C, the particle-enhanced immunoturbimetric method was used with reagents from Gentian (traceable to the international reference material ERM-DA471/IFCC).

### 2.3. Outcome Measures

The primary outcome measure was comparison of *relative* eGFR_crea_ and eGFR_cys_. Similar to other studies on mixed age populations [34,35] and children [36], absolute mean difference between eGFR_crea_ and eGFR_cys_ (|ΔeGFR_mean_|), expressed as a percentages, was used for the analysis instead of comparison with mGFR:

$$\left|{\Delta \mathrm{eGFR}}_{\mathrm{mean}}\right|=\left|\frac{{\mathrm{eGFR}}_{\mathrm{crea}}-{\mathrm{eGFR}}_{\mathrm{cys}}}{{\mathrm{eGFR}}_{\mathrm{crea}+\mathrm{cys}}}\right|$$

|ΔeGFR_mean_| ≥ 40% was considered significant, as larger discrepancy has been shown to be associated with low accuracy in eGFR_crea_ and/or eGFR_cys_ [34,35]. Proportion of |ΔeGFR_mean_| ≥ 40% was also calculated. The secondary outcome measure was concordance between eGFR_crea_ and eGFR_cys.,_ expressed in Lin’s concordance correlation coefficient (CCC). The tertiary outcome measure was proportion of consistent staging of renal failure between eGFR_crea_ and eGFR_cys_. Subgroup analyses were performed with regard to frailty according to Clinical Frailty Scale (CFS) [37,38] and three pre-defined age groups: <80 years, 80–89 years and ≥90 years.

#### 2.3.1. Lin’s Concordance Correlation

CCC is considered the most appropriate measure of *concordance* for methods measuring the *same continuous variable* [39]. Unlike other correlation measures, CCC also accounts for the vertical shift of the regression line from y = x which corresponds to perfect concordance [40]. Pearson correlation coefficient (*r*) measures the *correlation between different variables* and is inappropriate in concordance studies [41]. Like other correlation measures, CCC yields a value between -1 (perfect negative concordance) and 1 (perfect positive concordance), where interpretation of the result depends on the clinical context. A more conservative interpretation of CCC compared to other correlation measures has been proposed: >0.99 indicates very good concordance, 0.95–0.99 good, 0.9–0.95 moderate and <0.9 unsatisfactory concordance [42].

#### 2.3.2. Clinical Frailty Scale

While there is yet no general definition of frailty, there are several frailty scales in the field. One of the most common is CFS [43]. CFS grades *habitual* frailty on a nine-point scale based on nursing needs, activities of daily living (ADL), physical function and morbidity [37,38]. Habitual frailty is defined as functional status two weeks prior to the assessment [38]. In the development of the scale, patients <65 years of age and individuals with disabilities were excluded. CFS was developed to identify patients at high risk of adverse events (e.g., pressure ulcers and malnutrition) in a standardized way to enable patient-centered care [37,38]. The scale can be dichotomized, where CFS 1–4 correspond to non-frail (“robust”) and CFS 5–9 to frail [26,27,28]. CFS 9 means that the patient is terminally ill. In this study, frailty was graded during interdisciplinary conferences, attended by physicians, nurses, assistant nurses, occupational therapists and physiotherapists. The staff were not informed about the study’s outcome measures.

### 2.4. Statistical Analyses

Median and interquartile range (IQR) for continuous variables and percentages for categorical variables were used for descriptive purposes. eGFR_crea_ and eGFR_cys_ were compared using the Wilcoxon signed-rank test. Normally distributed groups were compared using ANOVA and non-normally distributed groups and ordinal data were compared using the Kruskal–Wallis test. Individual means were analyzed using one-sample t-test. Simple linear regression was used to test if CFS and age as independent variables significantly predicted |ΔeGFR_mean_|. Proportions were compared using the χ^2^-test or Fisher’s exact test. Data was considered normally distributed if the Shapiro–Wilk test ≥ 0.05. *p* < 0.05 was considered statistically significant. The confidence level for confidence intervals was set to 95%. Statistical analyses were performed using jamovi (version 1.6.18.0 for Mac), except for power calculations where SPSS (version 1.0.0.1508 for Mac) was used.

#### Power

A power calculation was performed a priori. In a large European study on a heterogeneous age cohort (*n* = 1200, median age = 63 years, SD = 20) |ΔeGFR_mean_| = 23% [34]. Thirteen subjects were required in our study to detect |ΔeGFR_mean_| ≥ 40% (α = 0.05, power 80%).

## 3. Results

### 3.1. Descriptive Statistics

A total of 111 patients were admitted during the study period. Cystatin C was not analyzed in 13 patients. Three patients had eGFR_cys_ > 90 mL/min/1.73 m^2^ and were excluded. In total, 95 patients fulfilled the inclusion criteria and were included in the final analysis. Six of the subjects were not graded according to CFS and six subjects were <65 years old. Patient characteristics are presented in Table 2.

A total of 76% of patients ≥ 65 years old were graded as frail, 16% had a BMI of <20 kg/m^2^ and 30% had renal impairment corresponding to stage 4 or 5. Frail patients were older than non-frail patients (*p* = 0.023). Frail patients were at a later stage of renal failure as estimated with both creatinine (*p* = 0.014) and cystatin C (*p* < 0.01). No statistically significant difference between frail and non-frail could be detected with regard to BMI *(p* = 0.49), proportion of thyroid treatment (*p* = 0.35), high-dose steroid therapy (*p* = 0.95) and length of stay (*p* = 0.93). Only one patient was terminally ill, i.e., CFS = 9.

The distribution of diagnoses is shown in Table 3. The most common were musculoskeletal, cardiological as well as urogenital and nephrological diagnoses. Osteoporosis-related fracture (including hip fracture) was the most common diagnosis (18%). Among cardiological diagnoses, heart failure was the most common (15%). In the urogenital and nephrological group, the most common diagnosis was urinary tract infection (8%). The distribution of diagnoses did not differ statistically significant between frail and non-frail patients (*p* = 0.19).

### 3.2. Outcome Measures

#### 3.2.1. Primary Outcome Measure

**Figure 1:**
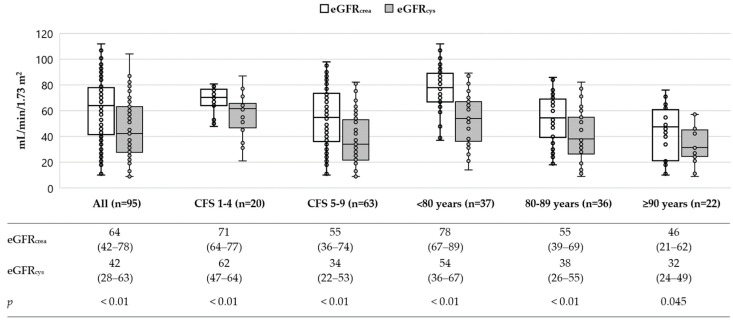
Box-and-Whisker plot showing median and range of eGFR estimated with the creatinine and cystatin C in geriatric and frail patients. The table below shows median and interquartile range (IQR). *p*-values were calculated with the Wilcoxon signed-rank test.

**Figure 2:**
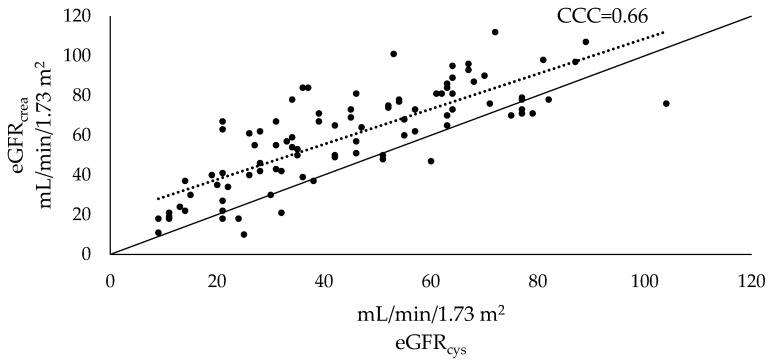
Paired estimates and CCC for eGFR_crea_ and eGFR_cys_ in geriatric patients. The dashed line corresponds to the regression line for eGFR_crea_ and eGFR_cys_. The solid line corresponds to perfect concordance (i.e., eGFR_crea_ = eGFR_cys_). eGFR_crea_ > eGFR_cys_ when a paired estimate is above the solid line, and vice versa.

Cystatin C estimated lower GFR than creatinine across the entire study population, as well as in all subgroups (Figure 1 and Figure 2).

|ΔeGFR_mean_| was greater in frail compared to non-frail patients (*p* < 0.01) (Table 4). Controlling for thyroid disease, high-dose steroid therapy and BMI < 20 kg/m^2^ did not affect the result significantly (*p* = 0.011). No statistically significant difference was detected between the age groups (*p* = 0.97). |ΔeGFR_mean_| exceeded 40% only in frail patients but was not statistically significant (*p* = 0.31). The proportion of |ΔeGFR_mean_| ≥ 40% was greater in frail compared to non-frail patients (*p* < 0.01). This was not observed between the age groups (*p* = 0.39).

Simple linear regression was used to test if CFS and age as independent variables significantly predicted |ΔeGFR_mean_| (Table 5). Age was a continuous variable in the regression (not stratified into different age groups). It was found that CFS significantly predicted |ΔeGFR_mean_|, i.e., |ΔeGFR_mean_| increased by 3.1–9.9 percentage points (95% CI) for each level in CFS (*p* < 0.01). Notably, age did not significantly predict |ΔeGFR_mean_| (*p* = 0.55).

#### 3.2.2. Secondary Outcome Measure

Figure 2 shows paired estimates of eGFR_crea_ and eGFR_cys_ with CCC as a concordance measure. The dashed line corresponds to the regression line for eGFR_crea_ and eGFR_cys_, and the solid line corresponds to perfect concordance (i.e., eGFR_crea_ = eGFR_cys_). CCC was 0.66 for the entire study population, 95% CI [0.55, 0.74] and did not reach 0.95 (i.e., cut-off value for good concordance) in any subgroup (Table 4).

#### 3.2.3. Tertiary Outcome Measure

The consistency regarding staging of renal failure with eGFR_crea_ and GFR_cys_, respectively, was 44% for the entire study population (Table 4). The consistency was lower in frail compared to non-frail *(p* = 0.035) patients. A statistically significant difference could not be detected between the different age groups (*p* = 0.84).

## 4. Discussion

The eGFR_cys_ estimated lower GFR than eGFR_crea_ across the entire study population as well as in all subgroups. This is in line with several other studies on elderly patients [12,14,15,16,18,20,23,24,29,44,45,46] published after SBU’s systematic review from 2012 [1]. A majority of these studies have also had mGFR as reference [12,14,15,16,18,20,23,24,29]. However, no study has been able to demonstrate which estimate that is preferable over the other. A majority still conclude that eGFR_crea+cys_ is favorable also in the elderly [12,14,15,16,18]. One Swedish and one Chinese study have been conducted comparing eGFR with mGFR in patients admitted to geriatric clinics [20,29]. In the Chinese study (*n* = 110), cystatin C generally estimated lower GFR than mGFR [29]. Both equations based on creatinine (CKD-EPI) and cystatin C (Tan, MacIsaac) had acceptable accuracy at mGFR > 60 mL/min/1.73 m^2^. However, only cystatin C-based equations (MacIsaac, Ma) had an acceptable accuracy at mGFR < 60 mL/min/1.73 m^2^. In the Swedish study (*n* = 108), all equations (Cockroft–Gault; MDRD4; CKD-EPI; CAPA; BIS2) had insufficient accuracy except Cockgroft–Gault [20]. Interestingly, in contradiction to this, Cockroft–Gault is generally not recommended in clinical practice due to its lack of accuracy [1].

In this study, discrepancy between eGFR_crea_ and eGFR_cys_ increased with increasing frailty. This could not be observed for increasing age. This indicates that increasing frailty rather than aging reduces accuracy of eGFR. However, the results must be interpreted with caution as it is a cross-sectional study with a relatively small sample size and conclusions about causality can therefore not be drawn [41].

The proportion of |ΔeGFR_mean_| ≥ 40% was 41% for the entire study population, 95% CI [32%, 51%], and 52% for frail patients, 95% CI [40%, 64%]. This complicates routine calculation of eGFR_crea+cys_ in this patient group as it is not valid when |ΔeGFR_mean_| exceeds 40% [34]. In contrast, |ΔeGFR_mean_| was 18%, 95% CI [16%, 21%], in a large European study on a heterogeneous age cohort (*n* = 1200, median age = 63 years) [34]. 

CCC did not reach 0.95 for all subjects or in any subgroup, which has been suggested as minimum value for good concordance [42]. However, this is the first study with CCC in this context, why significance assessment and comparison with other studies are not possible. There have been previous concordance studies on eGFR_crea_ and eGFR_cys_ in the elderly. They have, however, used intraclass correlation for the analyses, which is inferior to CCC for continuous variables [39,40,42]. We welcome more studies using CCC.

The staging of renal failure with eGFR_crea_ and eGFR_cys_, respectively, was consistent in almost 50% of the cases. This is in line with another study in elderly patients (*n* = 60), where mean consistency was 40–62% [47]. In our study, consistency was even lower in frail patients. However, there is an inherent uncertainty in equations for eGFR. According to international practice, an equation’s performance is assessed by *bias* and *accuracy* [1]. Bias refers to the mean or median difference between eGFR and mGFR, where >10% often is considered significant [1]. Accuracy refers to the proportion of estimates within a predetermined margin of error from mGFR [1]. A generally accepted proportion and margin of error is 80% and ±30%, respectively [1]. In summary, an equation is accepted even if there is a relatively large spread in up to 20% of the estimates, provided that the mean or median difference from mGFR is less than 10%. This has implications on drug dosing. In a Danish study (*n* = 338) of acutely ill elderly patients, 9.9–19.1% would have received a higher dose than recommended of at least one drug, depending on which equation that was used (CKD-EPI; BIS; Cockroft–Gault) [13]. Studies on adverse drug reactions or treatment failure in relation to usage of different equations have, to our knowledge, not been conducted. An additional difficulty with regard to drug dosing is that Cockroft–Gault is still recommended in clinical trials [48].

Several explanations for why cystatin C consistently estimate lower GFR in the elderly compared to creatinine have been presented. Muscle mass decreases with age, which masks deteriorated renal function due to lower creatinine levels [49,50,51,52]. A number of cross-sectional studies have shown correlation between sarcopenia and increasing creatinine-cystatin C ratio, i.e., the *sarcopenia index* [13,49,53,54,55]. No study has investigated the relationship between sarcopenia and accuracy of eGFR. Another theory is the *shrunken pore syndrome*, which causes shrinkage of pores in glomeruli (61). Large molecules, e.g., cystatin C (13 kDa), are then eliminated to a lesser extent, in contrast to small molecules, e.g., creatinine (0.12 kDa), which continue to be filtered freely [56]. This might explain why plasma levels of creatinine are not reduced until half of the nephrons are affected [57,58]. Consequently, toxins accumulate and cause a negative spiral with increased atherosclerosis and nephrosclerosis [31]. Several studies have been made to identify additional *non-GFR determinants* that affects creatinine and cystatin C levels, e.g., inflammation, diabetes, cancer and smoking. However, the results are contradictory and come mostly from cross-sectional studies [58,59]. It has been suggested that the improved accuracy in eGFR_crea+cys_ is due to each marker’s compensation for the other’s disadvantages [14]. In this study, we chose to control for the main non-GFR determinants suggested by SBU, i.e., thyroid disease, high-dose steroid therapy and underweight [1].

### Strenghts and Limitations

This is the first study to investigate the association between uncertainty in renal function estimation and CFS. A similar study (*n* = 55) has been done on psychiatric patients, but no correlation was detected between frailty and difference between eGFR_crea_ and eGFR_cys_ [44]. That study also used a different frailty scale (Rockwood Frailty Index) and had methodological differences compared ours. A strength of our study is that CFS was assessed during a multidisciplinary round. Inter-rater reliability for CFS is good in non-acute settings [25,37], in contrast to initial estimation in the emergency department where concordance has shown to be lacking [43]. Furthermore, the assessors of CFS were not aware of the outcome measures in this study, which reduces risk of bias. This is the first study using both LMR and CAPA in a geriatric context. CCC is rarely used in medical research despite its advantage when evaluating continuous variables and has never been used to evaluate consistency between different eGFR equations.

This study has several limitations. We were not able to analyze mGFR due to time-constraints. Instead, |ΔeGFR_mean_| was used as a surrogate measure of accuracy. |ΔeGFR_mean_| has indeed been evaluated in previous studies [34,35], but cannot be considered as an accepted measure of accuracy. The study was underpowered to detect |ΔeGFR_mean_| 40% in frail patients. Post hoc power was 28% to detect |ΔeGFR_mean_| ≥ 40% for all patients and 10% for frail patients. A total of 1924 patients would have been required to reach statistical power of 80% in the frail group, which is significantly more than predicted. This may be explained partly by a greater spread in the estimates (SD = 25% for all patients; SD = 27% for frail; SD = 18% for non-frail) compared to the study which served as basis for the power calculation a priori [12]. Furthermore, this is a single-center cross-sectional study and it is therefore not possible to draw conclusions about causality [41]. Prospective studies are necessary to answer this question.

This study was conducted in an acute geriatric setting. Acute illness is more likely to contribute to bias and is for that reason often used as an exclusion criterion in similar studies [15,16,19,22,23,24,29,49,50]. On the other hand, acute illness is a clinical reality and including such patients may give a better picture of daily practice.

Finally, this study cannot conclude whether eGFR_crea_ or eGFR_cys_ is preferable in this patient group since they were not compared to mGFR. Instead, the results from this study may provide a valuable background for the design and hypothesis in a future, prospective study where the estimates are compared with mGFR.

## 5. Conclusions

The results suggest that eGFR_crea_ and eGFR_cys_ differ significantly in geriatric and frail patients, where cystatin C estimates lower GFR compared to creatinine. Furthermore, this study suggests that frailty according to CFS may have greater impact than age on the accuracy of eGFR. The study cannot determine whether one of the GFR estimates is preferable to the other in these individuals. To answer this, studies comparing eGFR with mGFR are needed. Calculating eGFR_crea+cys_ has been shown to increase accuracy in other patients but may be difficult to introduce as routine practice in geriatric care, as the difference between the estimates was too large in almost 50% of the cases.

## Figures and Tables

**Table 1:** Stages of chronic renal failure according to Kidney Disease Outcomes Quality Initiative (KDOQI) [6].

Stage	eGFR	Micro- or Macroalbuminuria
1	≥90	Obligate
2	60–89	Obligate
3	30–59	Not obligate
4	15–29	Not obligate
5	<15	Not obligate

eGFR = estimated glomerular filtration rate [mL/min/1.73 m^2^]. Chronic renal failure is defined as persistent renal impairment >3 months [6]. Stages 1 and 2 require micro- or macroalbuminuria in addition to reduced eGFR. Stages 3–5 only require reduced eGFR.

**Table 2:** Patient characteristics. Continuous variables are reported as median (IQR). Categorical variables are reported as percentages.

	All (*n* = 95)	CFS 1–4 (*n* = 20)	CFS 5–9 (*n* = 63)
Age	84 (76–89)	80 (74–85)	85 (78–90)
CFS	6 (5–7)	3 (3–4)	6 (4–7)
Women	56%	60%	57%
Men	44%	40%	43%
BMI	24.4 (21.8–28.4)	25.0 (22.3–28.9)	24.2 (21.7–27.8)
Length of stay, days	6 (4–8)	6 (4–10)	7 (4–8)
Stage of renal failure eGFR_crea_	2 (2–3)	2 (2–2)	3 (2–3)
Stage of renal failure eGFR_cys_	3 (2–4)	2 (2–3)	3 (3–4)
Treatment for thyroid disease	17%	10%	19%
High-dose steroid therapy	8%	10%	10%

Abbreviations: BMI = body mass index [kg/m^2^]; CFS = Clinical Frailty Scale; eGFR_cys_ = eGFR with cystatin C; eGFR_crea_ = eGFR with creatinine. Patients assessed according to CFS were fewer than the total number of patients as six patients were <65 years old and another six patients were not assessed according to CFS. CFS 1–4 corresponds to non-frail (“robust”) patients and CFS 5–9 to frail patients. Staging of renal failure is according to KDOQI (no consideration was given to proteinuria and whether it was acute or chronic renal failure). High-dose steroid therapy was defined as >0.170 mg/kg/day prednisolone equivalents at admission.

**Table 3:** Distribution of diagnoses in the study population based on ICD-10 (*n* = 95).

Musculoskeletal (including fractures)	26%
Cardiological	17%
Urogenital and nephrological	15%
Lung diseases	8%
GI-related	6%
Neurological	5%
Neoplasms	4%
Mental and behavioral disorders	4%
Diabetes	2%
Infectious diseases	2%
Other	10%

**Table 4:** Outcome measures for comparison of creatinine and cystatin C to estimate renal function in geriatric and frail patients [95% CI].

	All (*n* = 95)	CFS 1–4 (*n* = 20)	CFS 5–9 (*n* = 63)	<80 Years (*n* = 37)	80-89 Years (*n* = 36)	≥90 Years (*n* = 22)
|ΔeGFR_mean_|	37% [32, 42]	23% [16, 31]	42% [35, 48]	38% [29, 46]	37% [29, 45]	34% [25, 44]
Proportion of |ΔeGFR_mean_| ≥ 40%	41% [32, 51]	18% [5, 36]	52% [40, 64]	32% [20, 49]	47% [32, 63]	45% [27, 65]
CCC	0.66 [0.55, 0.74]	0.65 [0.08, 0.72]	0.61 [0.47, 0.72]	0.49 [0.31, 0.64]	0.64 [0.46, 0.77]	0.80 [0.59, 0.91]
Consistent staging of renal failure	44% [35, 54]	65% [43, 82]	38% [27, 50]	41% [26, 57]	47% [32, 63]	46% [27, 65]

Abbreviations: |ΔeGFR_mean_| = absolute mean difference between eGFR_crea_ and eGFR_cys_ (|eGFR_crea_ − eGFR_cys_|/(eGFR_crea_ + eGFR_cys_)/2); CCC = Lin’s concordance correlation coefficient.

**Table 5:** Simple linear regression for CFS and age as independent variables for |ΔeGFR_mean_| [95% CI].

	CFS (*n* = 83)	Age (*n* = 95)
β-coefficient	0.065 ** [0.031, 0.099]	−0.002 [−0.007, 0.004]
*r* ^2^	0.15	0.004
Intercept	0.19 [−0.17, 0.21]	0.50 * [0.06, 0.94]

** *p* < 0.01; * *p* < 0.05; β-coefficient = slope.

## Data Availability

Raw data is available from the corresponding author on request.
